# Physician Partnerships and Adverse Outcomes among Breast Cancer Survivors: The Role of Adherence to Adjuvant Hormone Therapy

**DOI:** 10.1007/s11606-025-09962-0

**Published:** 2025-12-15

**Authors:** Chiu-Mei Yeh, Yiing-Jenq Chou, Shun-Ku Lin, Chia-Jen Liu, Nicole Huang

**Affiliations:** 1 Institute of Public Health, National Yang Ming Chiao Tung University, Taipei, Taiwan; 2 Division of Transfusion Medicine, Department of Medicine, Taipei Veterans General Hospital, Taipei, Taiwan; 3 School of Medicine, National Yang Ming Chiao Tung University, Taipei, Taiwan; 4 Department of Chinese medicine, Taipei City Hospital, Renai Branch, Taipei, Taiwan; 5 General Education Center, University of Taipei, Taipei, Taiwan; 6 Institute of Emergency and Critical Care Medicine, National Yang Ming Chiao Tung University, Taipei, Taiwan; 7 Institute of Hospital and Health Care Administration, National Yang Ming Chiao Tung University, Taipei, Taiwan

**Keywords:** breast cancer survivors, traditional Chinese medicine, care network, adherence to adjuvant hormone therapy, recurrence and mortality

## Abstract

**Background:**

Effective long-term care for breast cancer survivors requires collaboration among providers. Traditional Chinese medicine (TCM) physicians complement symptom management and quality of life. However, how collaboration between TCM physicians and Western medical oncologists/surgeons influences treatment adherence and outcomes remains unclear. Exploring this relationship may optimize interdisciplinary care.

**Objective:**

To explore the role of hormone therapy (HT) adherence in the relationship between physician partnership and survival outcomes in breast cancer survivors. We specifically assessed whether stronger connectedness between medical oncologists/surgeons and TCM physicians improves recurrence and mortality rates through improved HT adherence.

**Methods:**

We conducted a retrospective, population-based study using Taiwan’s National Health Insurance Research Database. We identified adult women with newly diagnosed breast cancer who underwent mastectomy from 2007 to 2016 and received at least 12 months of adjuvant HT. We constructed a physician-level care network for each patient where a tie between physicians was weighted by the number of patients sharing. Tie strength represented the connection between medical oncologists/surgeons and TCM physicians during the year before initial adjuvant HT. The primary endpoints were recurrence and mortality.

**Results:**

Among 50,671 patients (median age 52 years), only 40.6% maintained HT adherence. Patients whose oncologists/surgeons and TCM physicians had stronger connectedness showed significantly lower recurrence and mortality risks, with adjusted hazard ratios of 0.97 (95% confidence interval [CI] 0.92–1.03), 0.90 (95% CI 0.85–0.96), and 0.91 (95% CI 0.84–0.98) for low, medium, and high connectedness, respectively, compared to patients without TCM care. The effect of physician partnerships on progression-free survival was partially mediated by adherence to long-term adjuvant HT (12.2%).

**Conclusions:**

Enhanced connectedness between oncologists/surgeons and TCM physicians was associated with increased HT adherence and improved progression-free survival in breast cancer survivors. Our findings suggested that fostering interdisciplinary physician collaboration could benefit long-term outcomes for breast cancer patients via HT adherence.

**Supplementary Information:**

The online version contains supplementary material available at 10.1007/s11606-025-09962-0.

## INTRODUCTION

The health care needs of cancer survivors can be complex and always involve multiple care professionals. For instance, a 2020–2021 survey of 749 cancer patients at two major US cancer centers found that 70.2% had used at least one form of complementary and alternative medicine alongside conventional treatment.^1^ These therapies, including traditional Chinese medicine (TCM), are commonly adopted to help manage the side effects of conventional treatments, alleviate symptoms such as pain and fatigue, improve overall quality of life, and enhance their immune system function.^2–4^ However, some worry that cancer patients who rely heavily on TCM may delay or forgo standard Western treatments, potentially compromising their treatment outcomes. For example, a cross-sectional study in a Brazilian oncology clinic found that unregulated use of herbal medicines among cancer patients who received antineoplastic therapy, often without informing oncologists, may contribute to treatment nonadherence or delays and potentially compromise outcomes.^5^ Nearly half of patients reported using herbal medicines; however, 77% were unaware of possible herb-drug interactions, and 60% had not been asked about herbal use by their physicians. Among users, 37% faced risks of clinically significant interactions, including with chemotherapy agents. Studies also suggest that such interactions may lead to adverse side effects ranging from gastrointestinal distress and allergies to severe organ failure, including hepatotoxicity and nephrotoxicity.^6^ The antioxidants in herbal medicines may also reduce the effectiveness of radiotherapy and chemotherapy by neutralizing the oxidizing free radicals created by these treatments.^7,8^

According to the National Comprehensive Cancer Network guidelines, fostering collaboration between oncologists and other healthcare providers is critical to assuring quality of cancer care, improving treatment outcomes, and enhancing patient well-being.^9^ Therefore, strengthening relationships between western medicine and complementary and alternative medicine (CAM) providers could be key to maximizing the benefits and minimizing the possible harms of using CAM in cancer care. Studies have shown that stronger ties and connectedness between providers with different specialties significantly decrease avoidable hospitalization and increase patient survival among cancer patients.^10–12^ Our earlier study also showed that breast cancer survivors whose TCM doctors had the highest connectedness with Western physicians had the lowest rates of avoidable hospitalization and mortality.^13^ But our understanding of the mechanisms by which stronger patient-sharing relationships between physicians of different specialties lead to better patient outcomes among cancer patients is limited. Previous studies focused mostly on investigating how the relationship between specialists and primary care physicians influences patient outcomes,^11,14,15^ but none has explored whether and how the relationship between cancer specialists and TCM physicians influences cancer patient outcomes.

Medication adherence may play a significant role in explaining the influence of physician partnership and patient outcomes among breast cancer patients as many patients are advised to continuously use pharmacological therapies such as adjuvant hormone therapy (HT) for better long-term outcomes. Adjuvant HT has been proven effective at reducing recurrence and mortality for hormone-sensitive breast cancer.^16–18^ But some side effects of HT often lead to discontinuation and nonadherence to HT.^19–21^ Seeking care from TCM providers may reduce side effects and, hence, may improve HT adherence and outcomes.^22^ But some studies suggest otherwise. Instead of improving HT adherence, patients who sought TCM care did not have better HT adherence, if not worse.^23^ Moon et al. conducted a systematic review on HT adherence in breast cancer survivors and found that women using CAM had lower odds of persisting with aromatase inhibitor treatment, possibly related to beliefs about the efficacy of complementary therapies in managing side effects.^20^ We wonder whether partnership between TCM and cancer specialists may help to improve patient outcomes through improved adherence to adjuvant HT.

Therefore, this study aims to investigate whether stronger connectedness between medical oncologists/surgeons and TCM physicians within breast cancer patients’ care networks is associated with reduced recurrence and mortality, and whether adherence to adjuvant HT mediates this association.

## MATERIALS AND METHODS

### Data Sources

Our primary data source was the 2000–2018 National Health Insurance Research Database (NHIRD), which provides nationwide population-based enrolment and claims data for more than 23 million residents, representing more than 99% of Taiwan’s population.^24,25^ More specifically, the NHI Registry for Catastrophic Illness Patients (RCIP) records data on patients with major diseases, including cancer, who receive copayment exemption under the NHI program.^26^ The RCIP was used to identify the sample of breast cancer patients. The NHIRD also incorporates medical personnel and medical facility registries, which provide information on care providers. The National Cancer Registry and the Cause of Death Data were also linked. Cancer stages and pathological features are available in the National Cancer Registry. The Cause of Death Data provided information regarding the date and cause of death.

### Study Design and Sample

The study population included breast cancer patients registered in the RCIP, following the *International Classification of Diseases, Ninth Revision, Clinical Modification* major code 174. Only breast cancer patients whose pathology report, relevant laboratory findings, and imaging results have been reviewed and confirmed by cancer specialists are eligible to be registered in the RCIP and thus are exempted from cost-sharing obligations under the NHI program. We identified adult women (age ≥ 20 years) with newly diagnosed breast cancer who underwent mastectomy between January 1, 2007, and December 31, 2016. Due to the lack of biomarker information on estrogen receptor (ER) and progesterone receptor (PR), we could not precisely identify hormone-sensitive breast cancer patients. Therefore, we included only those receiving at least 12 months of adjuvant HT as a proxy for hormone-sensitive breast cancer. Adjuvant HT included both tamoxifen and aromatase inhibitors (e.g., anastrozole, letrozole, and exemestane).

### Patient Sharing Between TCM Physicians and Medical Oncologists/Surgeons

We constructed a physician-level care network for each breast cancer patient in which a tie between physicians was weighted by the number of shared patients.^27–31^ In Taiwan, breast cancer patients receive their primary treatment mainly from medical oncologists or surgeons after mastectomy, so we focused on exploring patient-sharing relationships between medical oncologists/surgeons and TCM physicians. The independent variable was tie strength, which describes the number of connections or patients shared between each pair of medical oncologists/surgeons and TCM physicians within a patient’s care network in the year preceding her initial adjuvant HT treatment.^11^ Moreover, we used the relative threshold of the strongest 10% of each physician’s ties as a cutoff to define a true link between the medical oncologists/surgeons and the TCM physicians in order to reduce the possibility of a spurious connection.^27^ Furthermore, because of the skewed distribution of tie strength, tie strength was constructed as a categorical variable and classified into three levels based on its tertiles.

### Adherence to Adjuvant HT

We used medication possession ratios (MPR) to evaluate patient adherence to adjuvant HT during the study period, which divided the patient’s “number of days’ supply held during HT period” by “prescription duration in HT period.”^18,23,32^ A binary adherence in adjuvant HT was constructed. A patient was classified as adherent in adjuvant HT if her MPR was 80% or higher.^33–36^

### Outcome Measures

The primary endpoint of the study was recurrence or mortality. Recurrence in a breast cancer patient is defined as (1) if the first disease progression after the remission period, as recorded in the National Cancer Registry, included recurrence information;^37,38^ or (2) if the patient received chemotherapy, radiation, or surgery three months or more after the end of initial primary treatment.^39^ Notably, mortality is defined as all-cause death recorded in the National Cause of Death Data. All patients were followed from HT initiation until recurrence, death, or the end of the year 2018.

### Statistical Analysis

Patients’ and physicians’ characteristics were summarized as frequencies (percentages) and medians (interquartile ranges [IQRs]). Group differences were compared using Pearson’s chi-squared test or Fisher’s exact test for categorical data, with the Mann-Whitney *U* test for continuous variables. Multivariable logistic regression controlling for patient and physician characteristics was performed to identify the effect of physician partnerships on adherence to adjuvant HT. In the survival analysis, the Kaplan–Meier method was used to calculate the cumulative incidence of recurrence and mortality, and a log-rank test was used to test the significant difference between groups. Cox proportional hazards regression models were used to determine the relationship between physician partnership and patient outcomes (i.e., recurrence and mortality).

Traditional and counterfactual event-based mediation analysis was applied. In the traditional approach, if the exposure coefficient decreased after adding the mediator to the model, then the existence of indirect mediating effects was confirmed.^40^ Counterfactual event-based mediation analysis was performed using the natural effect model proposed by Lange et al.^41^ This approach provides a tool to decompose the total effect of a given exposure into a natural direct effect and a natural indirect effect through the mediator. Confidence intervals (CIs) for mediation effects were obtained using the bootstrap method. In addition, sensitivity analyses were performed to determine the different cutoff values of MPR for adjuvant HT. Analyses were performed using SAS software, version 9.4 (SAS Institute Inc., Cary, NC) and STATA statistical software, version 15.1 (StataCorp, College Station, TX). The social network analyses and the mediation analysis were conducted using R Statistical Software, version 4.1.3. All the statistical tests were two-sided, and a *P* value of < 0.05 was defined as statistically significant.

## RESULTS

### Clinical Characteristics of the Study Population

We identified 93,418 patients newly diagnosed with breast cancer between January 1, 2007, and December 31, 2016. Of these, we excluded 15,053 patients who did not undergo mastectomy, 6,780 patients who had antecedent cancer, 10 patients who were aged < 20 years, 903 patients who had a follow-up period of less than one year, 19,861 patients who did not receive adjuvant HT for one year, and 140 patients who lacked basic information of their treating physicians (Supplemental Fig. 1). We enrolled 50,671 patients with a median age of 52 years (IQR 46–61 years). Among breast cancer survivors, 35.1% had a Charlson comorbidity index score of ≥ 2, 63.3% lived in urban areas, and 58.6% were low income. The baseline characteristics of breast cancer survivors who received adjuvant HT and had TCM physicians in their care network, as well as their level of tie strength, are listed in Table 1.

**Table 1:** Baseline Characteristics of Breast Cancer Survivors

Characteristics	Total	Without TCM provider	Tie strength between medical oncologist/surgeon and TCM doctor			*P* value
			Low	Medium	High	
	*n* = 50,671	*n* = 22,453	*n* = 11,551	*n* = 9,641	*n* = 7,026	
	*n* (%)	*n *(%)	*n *(%)	*n *(%)	*n *(%)	
Median age, years (IQR)	52 (46–61)	54 (46–63)	52 (45–61)	52 (45–61)	50 (45–58)	< 0.001
Age ≥ 50 years	30,382 (60.0)	14,195 (63.2)	6,791 (58.8)	5,652 (58.6)	3,744 (53.3)	< 0.001
Charlson comorbidity index						
0–1	19,165 (37.8)	9,163 (40.8)	4,189 (36.3)	3,248 (33.7)	2,565 (36.5)	< 0.001
1–2	13,730 (27.1)	5,519 (24.6)	3,314 (28.7)	2,971 (30.8)	1,926 (27.4)	
≥ 2	17,776 (35.1)	7,771 (34.6)	4,048 (35.0)	3,422 (35.5)	2,535 (36.1)	
Degree of urbanization						
Urban	32,082 (63.3)	14,265 (63.5)	7,435 (64.4)	6,060 (62.9)	4,322 (61.5)	< 0.001
Suburban	12,994 (25.6)	5,613 (25.0)	2,901 (25.1)	2,609 (27.1)	1,871 (26.6)	
Rural	3,003 (5.9)	1,384 (6.2)	677 (5.9)	522 (5.4)	420 (6.0)	
Unknown	2,592 (5.1)	1,191 (5.3)	538 (4.7)	450 (4.7)	413 (5.9)	
Income group						
Low income	29,690 (58.6)	13,319 (59.3)	6,804 (58.9)	5,637 (58.5)	3,930 (55.9)	< 0.001
Median income	13,875 (27.4)	5,926 (26.4)	3,181 (27.5)	2,687 (27.9)	2,081 (29.6)	
High income	6,183 (12.2)	2,753 (12.3)	1,371 (11.9)	1,185 (12.3)	874 (12.4)	
Unknown	923 (1.8)	455 (2.0)	195 (1.7)	132 (1.4)	141 (2.0)	
Stage						
0 and I	21,247 (41.9)	9,015 (40.2)	5,165 (44.7)	4,297 (44.6)	2,770 (39.4)	< 0.001
II	19,614 (38.7)	8,813 (39.3)	4,333 (37.5)	3,594 (37.3)	2,874 (40.9)	
III and IV	7,432 (14.7)	3,485 (15.5)	1,478 (12.8)	1,271 (13.2)	1,198 (17.1)	
Unknown	2,378 (4.7)	1,140 (5.1)	575 (5.0)	479 (5.0)	184 (2.6)	
Treatment						
Chemotherapy	31,768 (62.7)	13,865 (61.8)	6,940 (60.1)	5,918 (61.4)	5,045 (71.8)	< 0.001
Radiotherapy	18,767 (37.0)	7,799 (34.7)	4,478 (38.8)	3,713 (38.5)	2,777 (39.5)	< 0.001
Physician’s sex						
Male	45,213 (89.2)	19,942 (88.8)	10,119 (87.6)	8,647 (89.7)	6,505 (92.6)	< 0.001
Female	5,458 (10.8)	2,511 (11.2)	1,432 (12.4)	994 (10.3)	521 (7.4)	
Physician’s age, years						
< 45	17,895 (35.3)	7883 (35.1)	4,522 (39.1)	3,301 (34.2)	2,189 (31.2)	< 0.001
≥ 45	32,776 (64.7)	14,570 (64.9)	7,029 (60.9)	6,340 (65.8)	4,837 (68.8)	
Physician’s experience ≥ 5 years	48,703 (96.1)	21,527 (95.9)	10,955 (94.8)	9,348 (97.0)	6,873 (97.8)	< 0.001
Hospital ownership						
Private	34,238 (67.6)	15,191 (67.7)	8,023 (69.5)	6,545 (67.9)	4,479 (63.7)	< 0.001
Public	16,433 (32.4)	7,262 (32.3)	3,528 (30.5)	3,096 (32.1)	2,547 (36.3)	
Hospital geographic region						
North	27,312 (53.9)	13,294 (59.2)	6,685 (57.9)	4,830 (50.1)	2,503 (35.6)	< 0.001
Middle	14,647 (28.9)	5,404 (24.1)	3,134 (27.1)	3,209 (33.3)	2,900 (41.3)	
South	7,826 (15.4)	3,326 (14.8)	1,593 (13.8)	1,449 (15.0)	1,458 (20.8)	
East	852 (1.7)	412 (1.8)	135 (1.2)	149 (1.5)	156 (2.2)	
Islands	34 (0.1)	17 (0.1)	4 (0.0)	4 (0.0)	9 (0.1)	
Medical center status						
Medical center	26,140 (51.6)	11,555 (51.5)	5,786 (50.1)	4,935 (51.2)	3,864 (55.0)	< 0.001
Regional hospital	20,615 (40.7)	9,149 (40.7)	4,773 (41.3)	3,923 (40.7)	2,770 (39.4)	
District hospital	3,916 (7.7)	1,749 (7.8)	992 (8.6)	783 (8.1)	392 (5.6)	
Adherence to adjuvant hormone therapy	20,573 (40.6)	9,438 (42.0)	4,036 (34.9)	3,743 (38.8)	3,356 (47.8)	< 0.001

*TCM*, traditional Chinese medicine; *IQR*, interquartile range

### Adherence to Adjuvant HT Associated with Physician Partnership

Among breast cancer survivors, 40.6% had their MPR for adherence in adjuvant HT at or above the threshold (i.e., 80%). After adjusting for other characteristics, compared to those not seeking care from TCM physicians, patients who sought care from TCM physicians, but with low (adjusted odds ratio [OR] 0.74, 95% CI 0.70–0.77) or medium (adjusted OR 0.82, 95% CI 0.78–0.87) connectedness between their oncologists/surgeons and TCM physicians, had a significantly lower likelihood of achieving the threshold for adherence to adjuvant HT. Only patients whose medical oncologists/surgeons had a high connectedness with their TCM physicians were significantly more likely to do so (adjusted OR 1.08, 95% CI 1.02–1.14) (Table 2).

**Table 2:** Univariate and Multivariable Analysis of Factors Associated with Adherence to Adjuvant Hormone Therapy in Breast Cancer Survivors

Predictive variables	Univariate analysis		Multivariable analysis^b^	
	OR (95% CI)	*P* value	OR (95% CI)	*P* value
Tie strength^a^				
Without TCM provider	Reference		Reference	
Low tie strength	0.74 (0.71–0.78)	< 0.001	0.74 (0.70–0.77)	< 0.001
Middle tie strength	0.88 (0.83–0.92)	< 0.001	0.82 (0.78–0.87)	< 0.001
High tie strength	1.26 (1.19–1.33)	< 0.001	1.08 (1.02–1.14)	0.008
Age ≥ 50 years	1.05 (1.02–1.09)	0.004	1.06 (1.02–1.10)	0.006
Charlson comorbidity index				
0–1	Reference		Reference	
1–2	1.02 (0.97–1.06)	0.451	1.01 (0.96–1.06)	0.737
≥ 2	1.08 (1.04–1.13)	< 0.001	1.02 (0.97–1.07)	0.399
Degree of urbanization				
Urban	Reference		Reference	
Suburban	1.07 (1.02–1.11)	0.002	0.86 (0.82–0.90)	< 0.001
Rural	0.98 (0.91–1.06)	0.566	0.66 (0.61–0.72)	< 0.001
Income group				
Low income	Reference		Reference	
Median income	0.96 (0.92–1.00)	0.039	1.05 (1.01–1.10)	0.023
High income	0.92 (0.87–0.97)	0.003	1.05 (0.99–1.11)	0.115
Stage				
0 and I	Reference		Reference	
II	1.08 (1.03–1.12)	< 0.001	1.06 (1.01–1.10)	0.017
III and IV	1.20 (1.14–1.26)	< 0.001	1.14 (1.07–1.21)	< 0.001
Treatment				
Chemotherapy	1.05 (1.01–1.09)	0.015	1.00 (0.95–1.04)	0.848
Radiotherapy	1.04 (1.00–1.08)	0.048	1.07 (1.03–1.11)	0.001
Physician’s sex				
Male	1.19 (1.13–1.27)	< 0.001	1.09 (1.03–1.16)	0.006
Female	Reference		Reference	
Physician’s age ≥ 45 years	0.98 (0.94–1.01)	0.215	0.98 (0.94–1.02)	0.247
Physician’s experience ≥ 5 years	1.25 (1.14–1.37)	< 0.001	1.20 (1.09–1.33)	< 0.001
Hospital ownership				
Private	Reference		Reference	
Public	0.83 (0.80–0.86)	< 0.001	0.84 (0.80–0.87)	< 0.001
Hospital geographic region				
North	Reference		Reference	
Middle	2.11 (2.03–2.20)	< 0.001	1.87 (1.77–1.98)	< 0.001
South	1.83 (1.74–1.93)	< 0.001	2.30 (2.20–2.40)	< 0.001
East	2.57 (2.24–2.95)	< 0.001	2.58 (2.24–2.98)	< 0.001
Islands	0.85 (0.41–1.79)	0.675	0.78 (0.37–1.67)	0.527
Medical center status				
Medical center	Reference		Reference	
Regional hospital	1.01 (0.92–1.11)	0.873	1.14 (1.10–1.19)	< 0.001
District hospital	0.52 (0.43–0.63)	< 0.001	1.29 (1.17–1.42)	< 0.001
Clinic	0.77 (0.72–0.82)	< 0.001	0.60 (0.53–0.67)	< 0.001

*OR*, odds ratio; *CI*, confidence interval

^a^Tie strength between medical oncologist/surgeon and TCM doctor

^b^Adjusted for patient, hospital and physician characteristics

### Relationship Between Physician Partnership and Risk of Recurrence and Mortality

**Figure 1:**
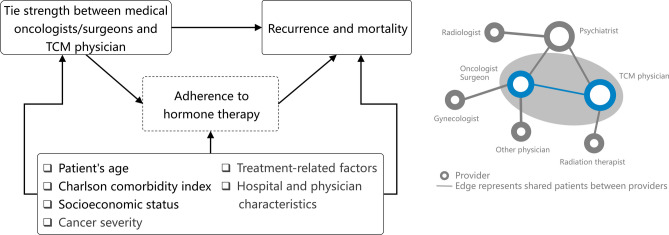
Path diagram of the relation between tie strength, adherence to long-term adjuvant hormone therapy, and recurrence and mortality.

**Figure 2:**
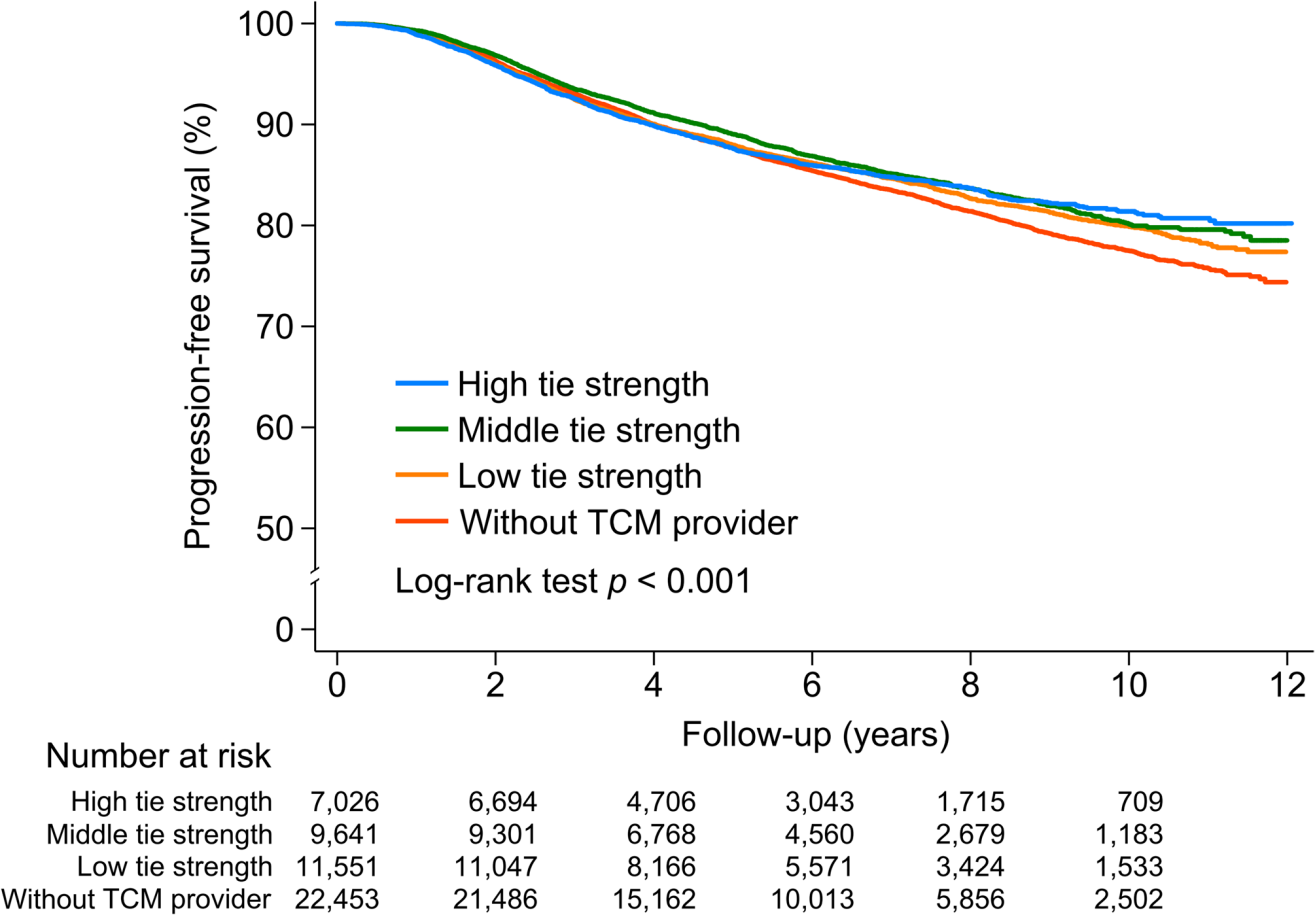
Kaplan–Meier survival curves in breast cancer survivors according to tie strength between medical oncologists/surgeons and TCM physicians

We assumed a causal relationship between the strength of physician connectedness, recurrence, and mortality through adherence in adjuvant HT (Fig. 1). The contribution of a binary mediator (adherence to adjuvant HT) to the effects of physician connectedness on recurrence and mortality was examined. Of the study sample, 7261 (14.3%) patients had a recurrence or died during the follow-up period. Among patients without a TCM provider, 3350 (14.9%) experienced these outcomes. In contrast, among those with a TCM provider, recurrence and mortality rates were 14.6% (*n* = 1681) in the low tie strength group and 13.4% (*n* = 1291) and 13.4% (*n* = 939) in both the medium and high tie strength groups, respectively. Five-year progression-free survival was 88.0% (95% CI 87.7–88.3%). The Kaplan–Meier curves show that progression-free survival was significantly better in patients whose medical oncologists/surgeons and TCM physicians had high connectedness (*P* < 0.001) (Fig. 2); the same was true among those who had adhered to adjuvant HT (*P* = 0.035) (Supplemental Fig. 2).

After adjusting for other characteristics, the connectedness between medical oncologists/surgeons and TCM physicians was significantly associated with recurrence and mortality after adjusting for patients’ and physicians’ characteristics. The adjusted hazard ratios (HRs) were 0.97 (95% CI 0.92–1.03), 0.90 (95% CI 0.85–0.96), and 0.91 (95% CI 0.84–0.98) for the low, middle, and high tie strength compared to patients without any TCM provider, respectively (Table 3). After including the presumed mediator, adherence to adjuvant HT, the association between physician connectedness and patient outcome remained unchanged.

**Table 3:** Factors for Recurrence and Mortality in Breast Cancer Survivors

Predictive variables	Univariate analysis		Multivariable analysis^b^			
			Without mediator		With mediator	
	HR (95% CI)	*P* value	HR (95% CI)	*P* value	HR (95% CI)	*P* value
Tie strength^a^						
Without TCM provider	Reference		Reference		Reference	
Low tie strength	0.93 (0.88–0.99)	0.024	0.97 (0.92–1.03)	0.372	0.98 (0.93–1.04)	0.594
Middle tie strength	0.87 (0.82–0.93)	< 0.001	0.90 (0.85–0.96)	0.002	0.91 (0.85–0.97)	0.004
High tie strength	0.91 (0.85–0.98)	0.011	0.91 (0.84–0.98)	0.010	0.90 (0.84–0.97)	0.008
Adherence to long-term adjuvant hormone therapy	0.95 (0.91–1.00)	0.036			0.86 (0.82–0.90)	< 0.001
Age ≥ 50 years	1.36 (1.29–1.42)	< 0.001	1.20 (1.14–1.26)	< 0.001	1.21 (1.15–1.27)	< 0.001
Charlson comorbidity index						
0–1	Reference		Reference		Reference	
1–2	1.03 (0.96–1.10)	0.394	1.00 (0.94–1.07)	0.912	1.00 (0.94–1.07)	0.919
≥ 2	1.95 (1.85–2.06)	< 0.001	1.32 (1.25–1.40)	< 0.001	1.32 (1.25–1.40)	< 0.001
Degree of urbanization						
Urban	Reference		Reference		Reference	
Suburban	1.13 (1.08–1.20)	< 0.001	0.99 (0.94–1.05)	0.768	0.99 (0.93–1.04)	0.631
Rural	1.38 (1.26–1.51)	< 0.001	1.04 (0.95–1.14)	0.392	1.03 (0.94–1.13)	0.587
Income group						
Low income	Reference		Reference		Reference	
Median income	0.77 (0.73–0.82)	< 0.001	0.88 (0.83–0.93)	< 0.001	0.88 (0.84–0.94)	< 0.001
High income	0.74 (0.68–0.79)	< 0.001	0.87 (0.80–0.94)	< 0.001	0.87 (0.80–0.94)	< 0.001
Stage						
0 and I	Reference		Reference		Reference	
II	2.00 (1.88–2.13)	< 0.001	1.93 (1.80–2.06)	< 0.001	1.93 (1.80–2.06)	< 0.001
III and IV	4.97 (4.66–5.30)	< 0.001	4.52 (4.19–4.87)	< 0.001	4.53 (4.21–4.89)	< 0.001
Treatment						
Chemotherapy	1.37 (1.30–1.44)	< 0.001	0.83 (0.78–0.88)	< 0.001	0.83 (0.78–0.88)	< 0.001
Radiotherapy	0.72 (0.68–0.75)	< 0.001	0.85 (0.80–0.89)	< 0.001	0.85 (0.81–0.89)	< 0.001
Physician’s sex						
Male	1.18 (1.09–1.28)	< 0.001	1.15 (1.06–1.26)	0.001	1.16 (1.06–1.26)	0.001
Female	Reference		Reference		Reference	
Physician’s age ≥ 45 years	0.92 (0.88–0.97)	0.001	0.96 (0.91–1.01)	0.097	0.96 (0.91–1.01)	0.086
Physician’s experience ≥ 5 years	0.85 (0.76–0.95)	0.004	0.95 (0.84–1.07)	0.388	0.96 (0.85–1.08)	0.465
Hospital ownership						
Private	Reference		Reference		Reference	
Public	0.87 (0.83–0.91)	< 0.001	0.91 (0.86–0.96)	< 0.001	0.90 (0.85–0.95)	< 0.001
Hospital geographic region						
North	Reference		Reference		Reference	
Middle	1.33 (1.26–1.40)	< 0.001	1.34 (1.25–1.43)	< 0.001	1.36 (1.28–1.46)	< 0.001
South	1.31 (1.23–1.40)	< 0.001	1.18 (1.11–1.25)	< 0.001	1.21 (1.14–1.28)	< 0.001
East	1.42 (1.20–1.67)	< 0.001	1.19 (1.01–1.41)	0.038	1.24 (1.05–1.47)	0.011
Islands	0.62 (0.20–1.90)	0.400	0.75 (0.24–2.36)	0.625	0.74 (0.24–2.32)	0.606
Medical center status						
Medical center	Reference		Reference		Reference	
Regional hospital	0.93 (0.82–1.05)	0.240	1.05 (0.99–1.10)	0.093	1.05 (1.00–1.11)	0.065
District hospital	0.70 (0.53–0.92)	0.009	1.06 (0.95–1.19)	0.301	1.07 (0.96–1.21)	0.227
Clinic	0.92 (0.84–1.00)	0.051	0.99 (0.87–1.12)	0.838	0.97 (0.86–1.10)	0.636

*HR*, hazard ratio; *CI*, confidence interval

^a^Tie strength between medical oncologist/surgeon and TCM doctor

^b^Adjusted for patient, hospital and physician characteristics

The traditional mediation analysis shows that the difference in hazards in recurrence and mortality between patient groups with different levels of physician connectedness slightly narrowed after adherence to adjuvant HT was included in the model. More specifically, the counterfactual event-based mediation analysis quantified the mediation effect of adherence to adjuvant HT on the relationship between physician connectedness, recurrence, and mortality in terms of natural direct and indirect HRs. 12.2% of the total effects of physician connectedness on patient outcomes (12.2%, 95% CI 2.6–34.3%) could be attributed to the indirect pathway mediated by adherence to adjuvant HT (Table 4). The total effect was then decomposed into a direct and an indirect HR, in which the indirect HRs of recurrence and mortality were 1.05 (95% CI 1.03–1.07), 1.03 (95% CI 1.02–1.04), and 0.99 (95% CI 0.98–1.00) for the low, middle, and high tie strength, respectively. Additionally, we conducted sensitivity analyses using different cutoff values of MPR (≥ 70%, 75%, and 85%) (Supplemental Table 1). Results remained robust, showing that adherence to adjuvant HT mediates the relationship between physician connectedness, recurrence, and mortality among breast cancer survivors.

**Table 4:** , Direct, and Indirect Effects of Tie Strength Between Medical Oncologist/Surgeon and TCM Doctor on Recurrence and Mortality

Predictive variables	Total effect		Direct effect^a^		Indirect effect^b^	
	HR (95% CI)	*P* value	HR (95% CI)	*P* value	HR (95% CI)	*P* value
Tie strength^c^						
Without TCM provider	Reference		Reference		Reference	
Low tie strength	1.02 (0.96–1.08)	0.497	0.97 (0.92–1.03)	0.372	1.05 (1.03–1.07)	< 0.001
Middle tie strength	0.93 (0.87–0.99)	0.028	0.90 (0.84–0.96)	0.002	1.03 (1.02–1.04)	< 0.001
High tie strength	0.90 (0.83–0.97)	0.004	0.91 (0.84–0.98)	0.010	0.99 (0.98–1.00)	0.027
Proportion mediated effect	100%		87.8% (65.7–97.4%)		12.2% (2.6–34.3%)	

*HR*, hazard ratio; *CI*, confidence interval. Results of mediation analysis adjusted for patient age, Charlson comorbidity index, degree of urbanization, income group, cancer stage, chemotherapy, and radiotherapy

^a^Effect of tie strength not explained by mediator

^b^Effect of tie strength through mediator

^c^Tie strength between medical oncologist/surgeon and TCM doctor

## DISCUSSION

Our study found that stronger connectedness between medical oncologists/surgeons and TCM physicians was associated with increased adherence to HT and reduced risks of recurrence and all-cause mortality among breast cancer survivors. Notably, the effect of physician partnerships on progression-free survival was partially mediated by adherence to long-term adjuvant HT (12.2%).

This finding aligns with the findings in other studies, indicating that strong connections between physicians positively impact patient outcomes. Previous studies have demonstrated that when different specialists maintain strong connections, patient treatment outcomes improve. Pollack et al. found that in the USA, greater connectedness among different specialties, such as primary care physicians and specialists, was associated with better diabetes management and lower hospitalization rates among patients diagnosed with locally advanced breast, prostate, or colorectal cancer.^10^ Another retrospective SEER-Medicare cohort study showed that collaboration between surgeons and medical oncologists improved all-cause mortality for patients with stage III colon cancer by ensuring timely and coordinated treatment plans.^11^ All these studies suggest that effective partnerships, not only within the same specialty but also across different fields and disciplines, play a crucial role in enhancing the quality of patient care and health outcomes. Our findings further reinforce such evidence, highlighting that interdisciplinary collaboration between cancer specialists and TCM doctors significantly improves outcomes for breast cancer survivors.

More importantly, our results also highlight the role of adherence to long-term adjuvant HT in explaining the influence of physician partnerships on reducing recurrence and mortality among breast cancer survivors. Adherence to adjuvant HT acts as an intermediary between physician partnerships and favorable outcomes for several reasons. In clinical settings, healthcare providers frequently interact through both formal and informal mechanisms, including patient referrals, shared patient management, and the exchange of clinical information. These interactions often occur when providers repeatedly care for the same patients, leading to familiarity with each other’s clinical practices, decision-making patterns, and communication styles. Over time, such shared experiences foster informal coordination and trust. First, stronger patient-sharing relationships may foster familiarity between medical oncologists/surgeons and TCM physicians, establishing a foundation of mutual trust. Mutual trust between physicians can be fostered through patient sharing, communication, and perceived competence, and lead to enhanced care coordination and interprofessional collaboration. Such trust-based relationships are particularly important in complex cases, promoting more cohesive and effective treatment planning across specialties.^42^ In our study, we propose a conceptual mediation pathway in which stronger physician connectedness facilitates better patient adherence to hormone therapy, which subsequently leads to improved outcomes such as reduced recurrence and mortality. Tie strength, operationalized as the number of shared patients between a medical oncologist or surgeon and a TCM physician, is widely recognized as a proxy for care coordination, trust, and mutual familiarity. In integrated care settings, increased patient sharing is associated with higher levels of informal communication and clinical alignment, which can support adherence to long-term therapies such as adjuvant HT. Consistent with this theoretical model, our mediation analysis found that adherence partially mediated the relationship between physician connectedness and outcomes, accounting for 12.2% of the total effect. While we acknowledge the limitations of causal inference in observational studies, the use of both traditional and counterfactual mediation analysis enhances the robustness of our findings.

Our findings also suggest that physician collaboration must be of sufficient strength or quality to yield clinical benefits. It is not merely the presence of a connection between TCM physicians and cancer specialists that matters, but rather the depth of their collaboration that influences treatment adherence and patient outcomes. Weak or infrequent connections may fail to provide the coordination and reinforcement necessary to support long-term hormone therapy adherence. This underscores the need for not only encouraging multidisciplinary engagement, but also strengthening the quality of physician partnerships within cancer care networks.

This trust could increase TCM physicians’ willingness to actively encourage their patients’ adherence to HT during their interactions with patients. Furthermore, less antagonistic attitudes toward other disciplines may reduce patients’ anxiety and foster a more positive care environment for breast cancer survivors in their care journey with HT therapy. By lowering the likelihood of HT discontinuation through collaborative care, physicians can create a stable and supportive environment that encourages patients to remain committed to their treatment. Our study also suggests that interventions from cancer specialists alone might not sufficiently improve adherence to HT. Close collaboration between oncologists/surgeons and TCM physicians could significantly contribute in this regard.

Second, a stronger connection may reduce potential antagonism or skepticism between Western and traditional practices. This collaborative care model likely enables physicians to work together effectively to manage side effects, provide consistent guidance, and promptly address patient concerns. A global consensus in oncology literature has highlighted that integrative oncology, which combines complementary therapies with conventional treatments, enhances symptom management and quality of life.^43^ A meta-analysis of 11 randomized controlled trials in elderly acute myeloid leukemia patients showed that integrated TCM and Western medicine yielded better outcomes: overall response rates (RR 1.23, 95% CI 1.13–1.33), complete remission rates (RR 1.38, 95% CI 1.15–1.65), and reduced adverse events such as myelosuppression, organ insufficiency, infections, and gastrointestinal discomfort.^44^ Rather than discouraging TCM involvement, oncologists/surgeons should take a more supportive stance toward complementary therapies, recognizing their potential to manage HT-related side effects. They should not discourage their patients’ access to TCM treatments, such as herbal medicine, acupuncture, or massage. These therapies may alleviate symptoms like fatigue, joint pain, and hot flashes, thereby improving patients’ tolerance for HT and promoting better adherence and treatment continuity. Ultimately, such collaborative support may improve patient prognosis.

Third, better connectedness may facilitate communication among cancer specialists, allowing TCM doctors to better understand patients’ current status and treatment progress, which may reduce risks of herb-drug interactions and, hence, decrease the likelihood of HT discontinuation. Overall, improved adherence, driven by strong physician partnerships, directly translates into better survival rates, lower recurrence, and an enhanced overall quality of life for breast cancer survivors.

Nonetheless, our study had some limitations. First, only baseline data on the mediator was available, which may have changed and fluctuated throughout the follow‐up. This could have amplified or diminished the mediator effect. Second, a major limitation of our study was the use of shared patients as a measure of physician partnership. While shared patient networks could indicate some degree of professional connection, they might not fully capture the depth or quality of collaboration between physicians. Shared patients might reflect logistical or geographic factors or even patient preferences rather than deliberate, coordinated partnerships. This measure might not entirely reflect the intentionality or strength of collaboration within a patient’s care network. Third, the recurrence and mortality risk factors tend to cluster, which may introduce some uncertainty in the precise estimates from the mediation analysis. Fourth, confounding by indication may be another major limitation. Due to data limitations, we could not adjust for important confounders fully, even though we have controlled for many patient and physician characteristics in our models. Fifth, due to the lack of biomarker information in the NHIRD, specifically hormone receptor status and HER2 status, we define hormone-sensitive breast cancer patients as those receiving at least 12 months of adjuvant HT. This approach may introduce misclassification bias, particularly among patients with HER2-positive tumors who may receive hormone therapy but follow more complex treatment regimens involving HER2-targeted agents. Moreover, recurrence and mortality outcomes may also be influenced by adherence to chemotherapy or radiotherapy, which were not the primary focus of this study. Finally, we limited the analysis to only patients with breast cancer who had undergone mastectomy and received hormone maintenance therapy, making it difficult to generalize the findings to all breast cancer survivors. Additionally, as this study was based solely on administrative claims data, it did not incorporate the perspectives of physicians or patients, which may provide important contextual understanding of the mechanisms driving treatment adherence and coordination.

Despite the limitations, this is the first study to explore whether connectedness between cancer specialists and TCM doctors can lead to better outcomes, and more importantly, identify the role of adherence to long-term adjuvant HT to explain the relationship between physician partnerships and patient outcomes among breast cancer patients. Our results suggest the importance of partnership among medical providers across different doctrines for improving breast cancer patients’ progression-free survival and potential influence of the postulated mediator, adherence to long-term adjuvant HT in this regard.

## Supplementary Information

Below is the link to the electronic supplementary material.

Supplementary Material 1 (DOCX 353 KB)

## Data Availability

The study is based on data from the National Health Insurance Research Database, provided by the Health and Welfare Data Science Center, Ministry of Health and Welfare (HWDC, MOHW).
