# Supplementary material for: Physician Partnerships and Adverse Outcomes among Breast Cancer Survivors: The Role of Adherence to Adjuvant Hormone Therapy
Source: J Gen Intern Med. 2025 Dec 15;41(7):1750–60. doi: 10.1007/s11606-025-09962-0 (PMC13176416; doi:10.1007/s11606-025-09962-0)

Supplementary Information

**Supplementary Table 1.** Results of Sensitivity Analysis Using Different Definitions of Adherence to Long-Term Adjuvant Hormone Therapy in Mediation Analysis

| **Tie strength^c^** | **Total effect** | | **Direct effect^a^** | | **Indirect effect^b^** | |
| --- | --- | --- | --- | --- | --- | --- |
|  | **HR (95% CI)** | ***P* value** | **HR (95% CI)** | ***P* value** | **HR (95% CI)** | ***P* value** |
| **MPR ≥ 70%** |  |  |  |  |  |  |
| Without TCM provider | reference |  | reference |  | reference |  |
| Low tie strength | 1.00 (0.94–1.06) | 0.920 | 0.98 (0.92–1.04) | 0.510 | 1.02 (1.00–1.03) | 0.020 |
| Middle tie strength | 0.92 (0.86–0.98) | 0.009 | 0.91 (0.85–0.97) | 0.003 | 1.01 (1.00–1.02) | 0.027 |
| High tie strength | 0.90 (0.84–0.97) | 0.006 | 0.91 (0.84–0.97) | 0.008 | 1.00 (0.99–1.00) | 0.112 |
| Proportion mediated effect | 100% | | 94.3% (83.1%–99.6%) | | 5.7% (0.4%–16.9%) | |
| **MPR ≥ 75%** |  |  |  |  |  |  |
| Without TCM provider | reference |  | reference |  | reference |  |
| Low tie strength | 1.01 (0.95–1.07) | 0.824 | 0.98 (0.92–1.04) | 0.452 | 1.03 (1.01–1.05) | < 0.001 |
| Middle tie strength | 0.92 (0.86–0.98) | 0.014 | 0.90 (0.85–0.96) | 0.002 | 1.02 (1.01–1.03) | 0.002 |
| High tie strength | 0.90 (0.83–0.97) | 0.005 | 0.91 (0.84–0.98) | 0.009 | 0.99 (0.98–1.00) | 0.040 |
| Proportion mediated effect | 100% | | 90.2% (74.0%–98.0%) | | 9.8% (2.0%–26.0%) | |
| **MPR ≥ 85%** |  |  |  |  |  |  |
| Without TCM provider | reference |  | reference |  | reference |  |
| Low tie strength | 1.05 (0.98–1.11) | 0.142 | 0.97 (0.91–1.03) | 0.274 | 1.08 (1.06–1.11) | < 0.001 |
| Middle tie strength | 0.94 (0.88–1.01) | 0.086 | 0.90 (0.84–0.96) | 0.001 | 1.05 (1.04–1.07) | < 0.001 |
| High tie strength | 0.89 (0.83–0.96) | 0.003 | 0.91 (0.84–0.98) | 0.010 | 0.98 (0.97–1.00) | 0.026 |
| Proportion mediated effect | 100% | | 82.1% (55.6%–97.1%) | | 17.9% (2.9%–44.4%) | |

HR, hazard ratio; CI, conﬁdence interval.

Results of mediation analysis adjusted for patient age, Charlson comorbidity index, degree of urbanization, income group, cancer stage, chemotherapy, and radiotherapy.

^a^Effect of tie strength not explained by mediator

^b^Effect of tie strength through mediator

^c^Tie strength between medical oncologist/surgeon and TCM doctor

**Supplementary Fig. 1.** Patient Selection Flowchart

**
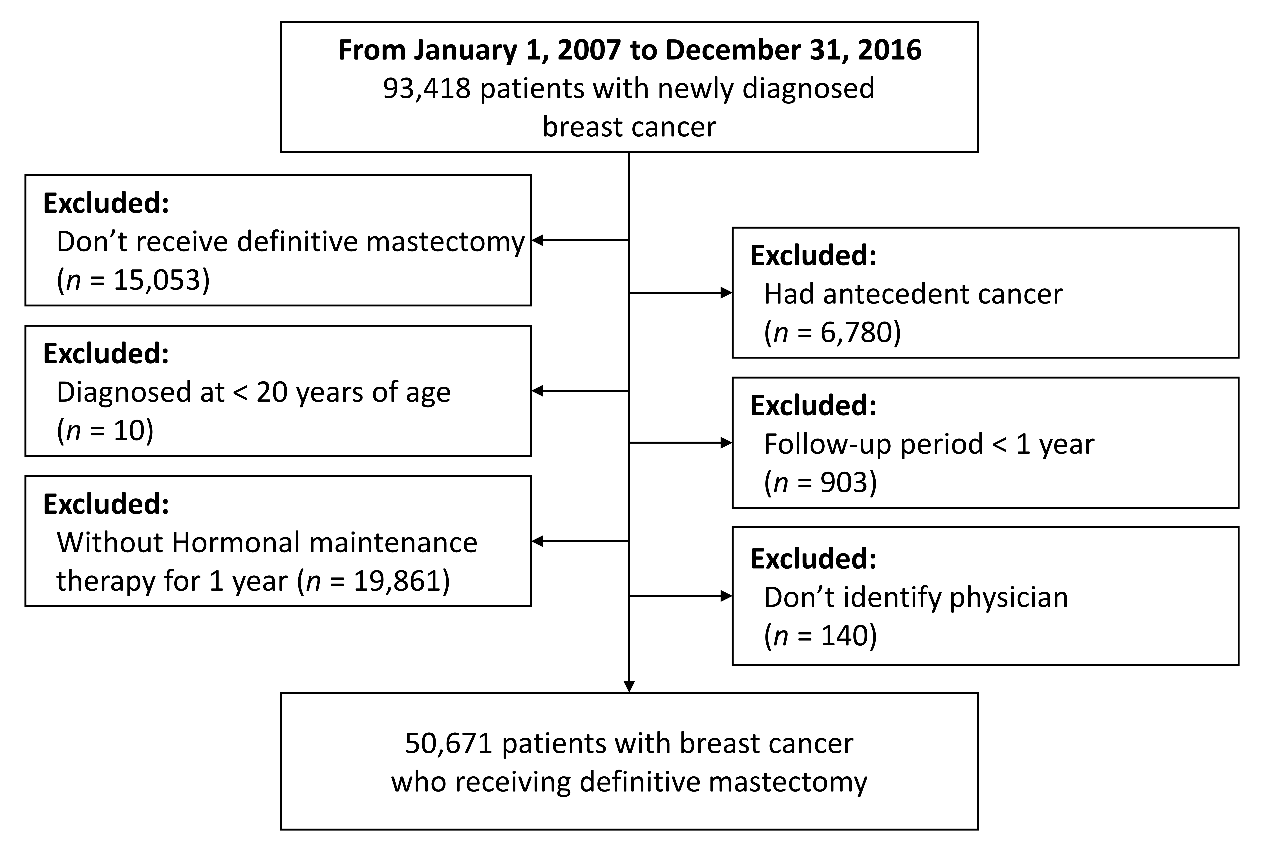
**

**Supplementary Fig. 2.** Long-Term Follow-Up of Progression-Free Survival for Adherence vs. Non-adherence to Long-Term Adjuvant Hormone Therapy among Breast Cancer Survivors


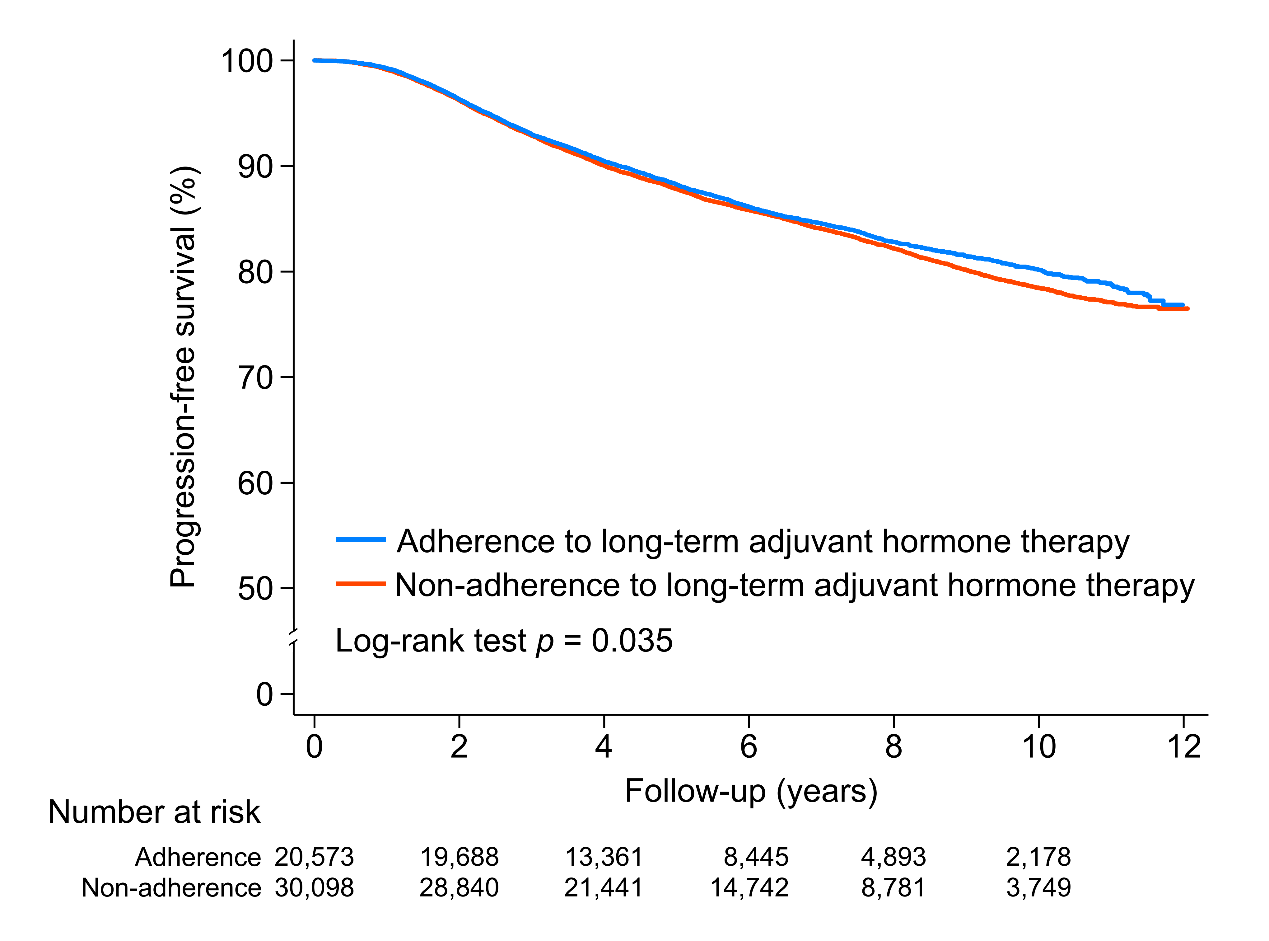

Supplement: Supplementary file 1 — Supplementary Material 1 (DOCX 353 KB) [file 11606_2025_9962_MOESM1_ESM.docx]
